# Energy metabolism disorders in migraine: triggers, pathways, and therapeutic repurposing

**DOI:** 10.3389/fneur.2025.1561000

**Published:** 2025-04-02

**Authors:** Wen-xiu Sun, Ting-yan Chen, Mao-mei Song, Ying-jie Gao, Sui-yi Xu

**Affiliations:** ^1^Department of Neurology, Headache Center, The First Hospital of Shanxi Medical University, Taiyuan, Shanxi, China; ^2^Department of Neurology, Headache Center, Tianjin First Central Hospital, Tianjin, China

**Keywords:** insulin resistance, mitochondrial dysfunction, antioxidant defenses, oxidative stress, lipid metabolism

## Abstract

Many migraine triggers, such as stress, sleep deprivation, fatigue, strenuous exercise, and fasting, are potentially linked to disturbances in brain energy metabolism, mitochondrial function, and oxidative stress. Alongside efforts to avoid modifiable factors, prophylactic migraine treatments that target brain energy metabolism have garnered increasing attention. However, the current evidence supporting the use of energy-modulating drugs in migraine treatment guidelines remains weak. This narrative review explores the relationship between energy metabolism and cortical spreading depression susceptibility, metabolic alterations in migraine (including glucose and insulin metabolism, insulin resistance, lipid metabolism, and energy metabolism imaging markers), oxidative stress and antioxidant defenses, mitochondrial dysfunction, and the role of energy metabolism-targeted medications in migraine management. Nutrients may help improve mitochondrial function, thereby alleviating brain energy metabolism deficits and oxidative stress in migraine.

## Introduction

Among 369 diseases analyzed across 204 countries, headache disorders ranked 14th in disability-adjusted life years (DALYs) for the general population and were positioned as the second leading cause of DALYs in females aged 15–49 years (1). Migraine constitutes the second leading cause of health loss measured by years lived with disability (YLD) among all diseases in the general population, surpassing the aggregate burden of all other neurological disorders combined ‌ (2). Of note, migraine has been the leading cause of DALYs among youths and young adults in East Asia over the past 30 years (3). Numerous external environmental and internal factors are known to trigger migraine. External factors include temperature fluctuations, bright lights, loud noises, and strong odors, while internal triggers encompass anxiety, emotional stress, insomnia, specific foods, and inadequate blood glucose supply (4). A growing body of evidence points to a significant link between migraine and disruptions in brain energy metabolism (5). Many migraine triggers, such as stress, sleep deprivation, fatigue, strenuous exercise, and fasting, are potentially related to disturbances in brain energy metabolism, mitochondrial function, and oxidative stress (6). Clinical studies have also identified elevated levels of inflammatory markers and oxidative stress biomarkers in individuals with migraine (7). It has been suggested that the brain energy deficit-mitochondrial-oxidative stress axis may represent a key pathway in migraine pathogenesis (8). As a result, prophylactic treatments for migraine that target brain energy metabolism, including riboflavin, coenzyme Q10, alpha-lipoic acid (ALA), and ketogenic diet (KD), have gained increasing attention (Table 1).

**Table 1:** Prophylactic treatments for migraine target brain energy metabolism.

Author/date	PMID	Dose/Duration	Patient/Model	Outcome
Riboflavin				
Schoenen et al. (76)	9,484,373	400 mg/day, 3 months	Adults with migraine (*n* = 55)	↓ Migraine frequency and attack duration
Bruijn et al. (84)	20,974,610	50 mg/day, 40 weeks	Children with migraine (*n* = 42)	No significant difference in migraine attack frequency
Nazıroğlu et al. (81)	25,492,827	100 mg/day, 10 days	Migraine rat model (*n* = 30)	Reduced oxidative damage and protected against glyceryl trinitrate-induced headaches
Das and Qubty (82)	33,189,027	100 or 200 mg/day, 3 months	Children and adolescents (*n* = 42)	↓ Migraine frequency, intensity and duration
Coenzyme Q10				
Rozen et al. (93)	11,972,582	150 mg/day, 3 months	Adults with migraine (*n* = 32)	↓ Migraine frequency and headache-days
Sándor et al. (92)	15,728,298	300 mg/day, 3 months	Adults with migraine (*n* = 42)	↓ Migraine frequency, headache-days and days-with-nausea
Slater et al. (96)	21,586,650	100 mg/day, 12 weeks	Children and adolescents (*n* = 120)	No difference in outcomes between the CoQ10 and placebo groups
Gaul et al. (86)	25,916,335	150 mg/day, 3 months	Adults with migraine (*n* = 130)	↓ Migraine frequency and intensity
Shoeibi et al. (94)	27,670,440	100 mg/day, 3 months	Adults with migraine (*n* = 80)	↓ Migraine frequency, duration and severity
ALA				
Cavestro et al. (105)	28,976,801	800 mg/day, 6 months	Patients with migraine (*n* = 32)	↓ Migraine frequency and treatment-days
Rezaei Kelishadi et al. (104)	34,105,866	600 mg/day, 3 months	Women with episodic migraine (*n* = 92)	↓ Oxidative stress and inflammatory markers
Kelishadi et al. (103)	34,997,178	600 mg/day, 12 weeks	Women with episodic migraine (*n* = 92)	↓ Migraine frequency, duration and severity
Puliappadamb et al. (106)	37,563,914	300 mg/day, 12 weeks	Adolescents with migraine (*n* = 60)	↓ Migraine frequency, duration and severity
KD				
Di Lorenzo et al. (111)	25,156,013	KD or SD, 6 months	Overweight female migraineurs (*n* = 96)	↓ Migraine frequency and headache-days
Merlino et al. (116)	37,209,426	KD, 3 months	Adults with migraine (*n* = 70)	↓ Migraine frequency and ↑sleep quality
Tereshko et al. (119)	37,501,109	KD, 3 months	High-frequency episodic and chronic migraine (*n* = 60)	↓ Migraine frequency and severity

PMID, PubMed unique identifier; ALA, Alpha-lipoic acid; KD, Ketogenic diet; SD, Standard low-calorie diet.

## Energy metabolism and cortical spreading depression susceptibility

Cortical spreading depression (CSD) is a neurophysiological phenomenon characterized by the strong depolarization of regional neurons or glial cells, spreading along the cortex to neighboring regions at a rate of 3–5 mm/min, ultimately leading to inhibition of neural activity (9). CSD is closely associated with migraine aura but is also observed in cerebral ischemia, epilepsy, and traumatic brain injury (10). The mechanisms underlying CSD involve an imbalance in the brain’s ionic homeostasis and disruptions in energy metabolism. Noninvasive detection of CSD in rats using near-infrared spectroscopy suggests that O₂ transport from blood to mitochondria is restricted, leading to reduced oxygen utilization during CSD (11). In addition to migraine-related CSD, brief needling of the frontal cortex has been shown to induce CSD, resulting in a sustained increase in cerebral metabolic rate of oxygen and a decrease in basal cerebral blood flow (12). CSD is associated with significant cerebral vasoconstriction (13), which triggers neuronal swelling and activation of meningeal trigeminal nerve endings and the trigeminal vascular system, ultimately contributing to migraine development (14–16). Cerebral glycogen deficiency or sleep deprivation leads to elevated extracellular potassium and glutamate concentrations (17), lowering the threshold for CSD. In summary, an inadequate energy supply increases susceptibility to CSD (18).

## Metabolic flux changes in migraine

### Glucose/insulin metabolism and insulin resistance

**Figure 1:**
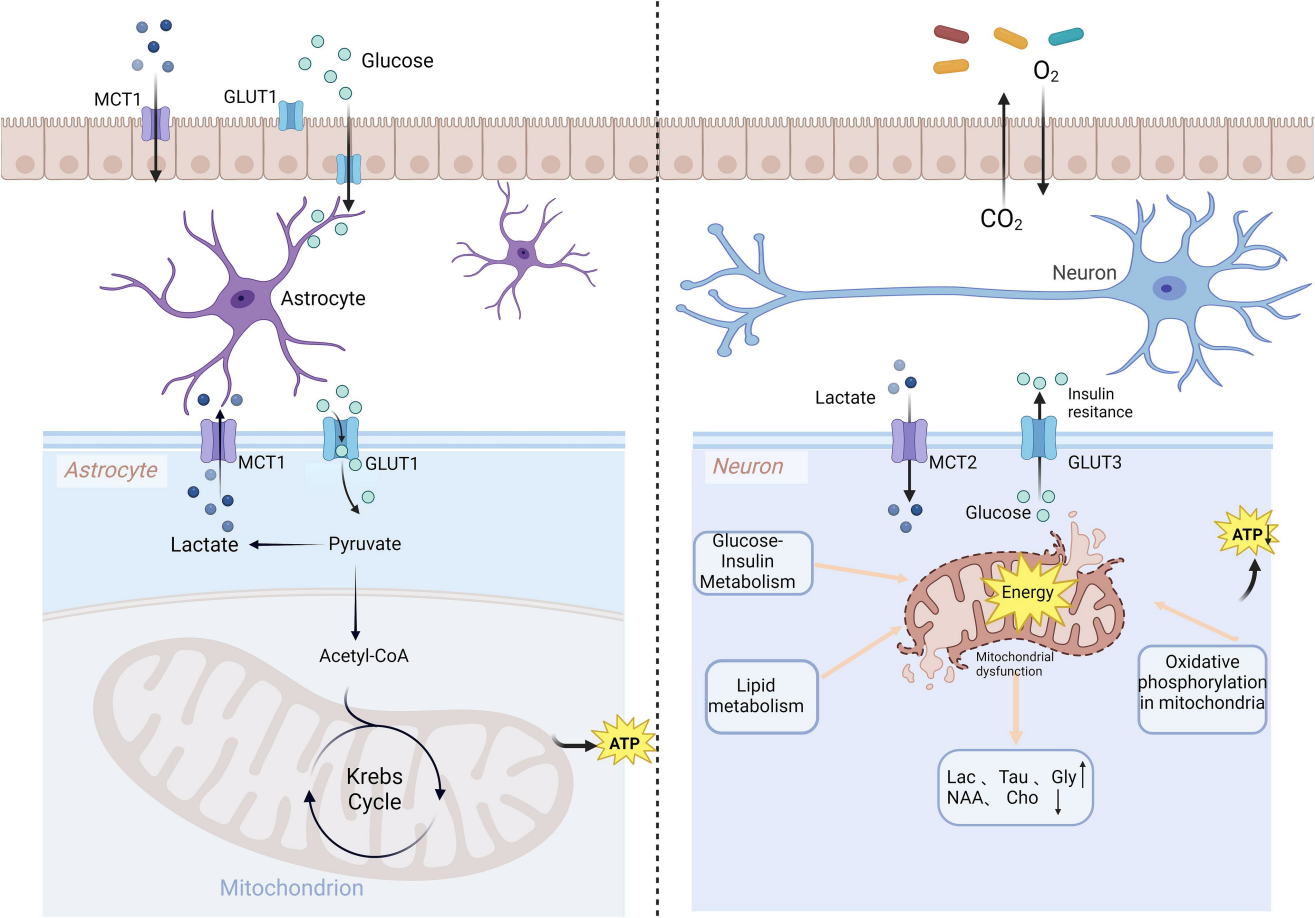
Brain metabolic disorders and migraine. ATP generation from glucose is the primary process of brain energy production and involves three key steps: glycolysis, the Krebs cycle, and oxidative phosphorylation. Disruptions in glucose-insulin metabolism, lipid metabolism, and oxidative phosphorylation impair mitochondrial function, leading to the development of migraine. MCT, monocarboxylate transporter; GLUT, glucose transporter; ATP, adenosine triphosphate; Lac, lactate; Tau, taurine; Gly, glycine; NAA, N-acetylaspartate; Cho, choline.

The brain relies on glucose as its primary energy substrate. Key pathways for energy production closely related to migraine include the synthesis of adenosine triphosphate (ATP) through glycolysis, the tricarboxylic acid cycle, and oxidative phosphorylation (Figure 1). Investigating cerebral glucose uptake using 18FDG-positron emission tomography (PET) and visual evoked potentials between patients with interictal migraine without aura and healthy volunteers (19) has identified areas of increased neuronal activation-resting glucose uptake ratios in the optic cortex. Elevated plasma glucose levels have been observed in patients with migraine during attacks (20). Disruption of cerebral metabolic homeostasis is thought to be a cornerstone of migraine pathophysiology (19, 21). Migraine has been linked to glycolysis, gluconeogenesis, and riboflavin metabolic pathways through proteomic and metabolomic study (22). Insulin resistance may be a critical metabolic link between migraine and its comorbidities (23), as insulin regulates mitochondrial signaling pathways (24). Chronic migraine has been associated with diabetes mellitus, insulin resistance, and metabolic syndrome (25). Case–control studies have demonstrated the presence of insulin resistance in individuals with migraine, a condition comparable to pre-diabetes (26, 27). Triglyceride-glucose, an index used to assess insulin resistance, has been shown to have a linear association with migraine (28). Furthermore, a recent study revealed significant genetic correlations between fasting insulin, glycosylated hemoglobin, and migraine (29), offering new insights into its pathogenesis at the genomic level. A blood biomarker study also found that triglyceride-glucose, C-reactive protein and phosphorus could guide treatment and preventive interventions for metabolic migraine (5).

### Lipid metabolism

Lipids serve as a major form of energy storage and as precursors for molecules involved in inflammation and pain signaling. However, the mechanisms through which lipid metabolism contributes to migraine pathology remain incomplete. Some findings suggest that migraine is associated with alterations in the metabolism of high-density lipoprotein isoforms rather than with general dyslipidemia (30). Changes in the activity of fatty acid elongation enzymes in plasma and cerebrospinal fluid may play an important role in the development of chronic migraine. Preliminary studies indicate that abnormal lipid metabolism in chronic migraine correlates with disruptions in energy homeostasis (31).

### Neuroimaging markers of energy metabolism

Neuroimaging studies suggest that patients with migraine exhibit abnormal brain energy metabolism and altered functional connectivity (32). Magnetic resonance spectroscopy (MRS) is a non-invasive method used to study *in vivo* tissue metabolism by measuring certain atomic nuclei, primarily hydrogen (1H). A study utilized 1H-MRS and resting-state functional magnetic resonance imaging to investigate metabolic changes throughout the migraine cycle and their relationship to functional connectivity. This study found that N-acetylaspartate (NAA)/total creatine (tCr) and choline (Cho)/tCr levels changed in a time-phase-dependent manner in migraine (33). PET studies suggest dysfunction in the thalamocortical pathways in patients with chronic migraine, potentially contributing to migraine chronicity (34). Patients with Migraine showed increased concentrations of lactate, taurine, tCr, and glycine, as well as decreased levels of NAA and total choline, as measured by 1H-MRS (35). Migraine attacks are associated with neuronal mitochondrial dysfunction and abnormal levels of glutamate and gamma-aminobutyric acid (GABA). The decrease in NAA may be a consequence of mitochondrial dysfunction and abnormal energy metabolism (36).

### Oxidative stress and antioxidant defense in migraine

A study using a migraine rat model revealed increased malondialdehyde levels in the cortex and trigeminal ganglion, along with pro-injurious effects of reactive oxygen species (ROS) on the brain via transient receptor potential A1 (16). Clinical studies examining oxidative stress biomarkers in individuals with migraine have found that the number of monthly headache days was negatively correlated with serum concentrations of two antioxidant enzymes, catalase and superoxide dismutase, and total antioxidant capacity while being positively correlated with serum levels of glutathione peroxidase 1, nitric oxide, and malondialdehyde (37–39). Serum analysis of 32 patients with high-frequency migraine revealed abnormally low levels of alpha-lipoic acid (ALA) and lactate, along with abnormally high levels of peroxides in 46.9% of the patients, further supporting the role of oxidative stress and metabolic alterations in the pathophysiology of migraine (40).

Oxidative stress primarily refers to the process of oxidative damage resulting from an imbalance between oxidative and antioxidant systems in cells and tissues, leading to the accumulation of free radicals and ROS (41). Oxidative damage to lipids, amino acids, and proteins impairs mitochondrial function, triggering a vicious cycle of ROS generation and cellular damage (42). Clinical studies have shown elevated serum lipid peroxide levels in patients with migraine (43). Individuals with chronic migraine tend to have lower antioxidant capacity and higher oxidative stress levels (44). Pharmacological studies suggest that alleviating migraine nociceptive sensitization may be achieved through enhancing endogenous antioxidant defense systems and targeting anti-inflammatory pathways (45, 46).

### Mitochondrial dysfunction in migraine

The mitochondrial oxidative phosphorylation system is central to cellular metabolism. The respiratory chain within the inner mitochondrial membrane consists of enzyme complexes (I, II, III, IV, and V), which form a major structural and functional component of mitochondria (47). The electron transport chain catalyzes the phosphorylation of ADP to ATP. Under abnormal metabolic conditions, such as hypoxia, ROS are primarily produced by complexes I and III. The generated ROS, in turn, induces mitochondrial DNA damage, contributing to oxidative stress (42, 48). 31P-MRS is a reliable, non-invasive tool for the *in vivo* assessment of mitochondrial function. Significantly lower levels of high-energy phosphates, such as ATP and PCr, as well as reduced oxidative phosphorylation, have been observed in patients with migraine and are closely linked to insufficient mitochondrial energy reserves (49, 50). Individuals with migraine exhibit significantly higher blood lactate levels and lower levels of NAA, along with decreased activities of nicotinamide adenine dinucleotide dehydrogenase, citrate synthase, and cytochrome c oxidase, suggesting a systemic impairment of mitochondrial function (51–53). Additionally, mitochondria are connected to the endoplasmic reticulum and co-regulate oxidative stress and mitochondrial autophagy (41). The PINK1/parkin pathway is a key mediator of mitophagy (54), effectively removing damaged mitochondria and avoiding excessive ROS production (55–58). Alterations in autophagic flux have been shown to modulate oxidative stress and ROS formation (57). Mitophagy plays a critical role in conditions such as stroke, cerebral ischemia–reperfusion injury, and neurodegenerative diseases (59–62). Autophagy dysfunction has been linked to central sensitization in a nitroglycerin-induced chronic migraine model in mice (63). However, the relationship between mitophagy and migraine remains incompletely understood, and further investigation is needed to clarify how mitochondrial quality control interacts with oxidative stress in migraine.

Animal studies have shown that migraine is associated with mitochondrial dysfunction. ATP levels and basal oxygen consumption rates were reduced in the fasting group, and adenylate-activated protein kinase phosphorylation levels were decreased (64). Case studies suggest that 61% of patients with mitochondrial disease experience migraine-like headaches (65). Similarly, online questionnaires have found a high prevalence of migraine-like headaches among patients with mitochondrial disease (66). 31P-MRS has revealed defective energy metabolism in the brain and muscles of individuals with familial hemiplegic migraine, further suggesting mitochondrial dysfunction in this genetic group (67). In addition, muscle mitochondrial DNA deletions have been reported in patients with migraine, marking the first instance of mitochondrial DNA deletion linked to migraine. Most mitochondrial proteins are involved in ATP synthesis and the Krebs cycle. Mitochondrial proteomic analysis has demonstrated that the m.8296A > G variant results in differential expression of nuclear-encoded proteins involved in energy metabolism, suggesting a connection between mitochondrial dysfunction and energy metabolic diseases (68). Nutrients may help improve mitochondrial function, thereby alleviating brain energy metabolism deficits and oxidative stress in migraine (69). Mitochondrial DNA methylation plays a crucial role in the regulation of mitochondrial genes, and directed changes in the mitochondrial genome and DNA may influence cellular energy dynamics (24). A study using a migraine rat model revealed that alterations in mitochondrial dynamics inhibit mitochondrial biosynthetic signaling in trigeminal ganglion neurons (70). In a nitroglycerin-induced migraine rat model, sodium valproate demonstrated protective effects on mitochondrial energy metabolism and biosynthesis (71). A recent study found that mitochondrial damage occurs in the thalamus of mice with chronic migraine and may be implicated in central sensitization (72). Serum analysis of patients with high-frequency migraine demonstrated significant abnormalities in most markers of mitochondrial function (40). However, existing animal models of migraine primarily mimic secondary migraine-like headaches. Understanding the correlation between biomarkers of energy metabolism disorders and headache severity is critical for translating basic research into clinical practice.

### Energy metabolism therapy in migraine

Oxidative stress and brain energy metabolism disorders play a crucial role in the pathogenesis of migraine (73). Antioxidant drugs and nutrients hold promise as potential prophylactic treatments for migraine (44). Current migraine prophylaxis targeting mitochondrial function and energy metabolism includes riboflavin, coenzyme Q10, lipoic acid, and ketogenic ketones. However the level of evidence-based medicine supporting these treatments remains limited.

Riboflavin, a water-soluble member of the vitamin B family, plays an important role in combating oxidative stress and maintaining mitochondrial function (74). It is recommended by the Canadian Headache Society for migraine prophylaxis (75). High doses of riboflavin (400 mg) have demonstrated potential effectiveness for adult migraine prevention (76). Riboflavin promotes energy production, protects the brain from oxidative stress (77), and has been shown to be effective in clinical trials for migraine prevention (69). It is involved in key metabolic pathways, including the tricarboxylic acid cycle, oxidative phosphorylation, and the metabolism of amino acids, fatty acids, and nucleotides (78, 79). Riboflavin transporters and flavin adenine dinucleotide-forming enzymes form a coordinated network to ensure cellular homeostasis (79). Riboflavin’s therapeutic potential has been demonstrated in both experimental and clinical migraine studies (80). A migraine rat model study found that selenium and riboflavin support the protective effects of the brain’s antioxidant system, and their combined use may offer enhanced protection (81). Studies on the efficacy of riboflavin in preventing migraine in children and adolescents indicate a reduction in migraine attacks; however, further research is needed to understand the relationship between dose, duration of administration, and the frequency and duration of migraine attacks (82–85). In adults, riboflavin’s efficacy in preventing migraine is classified as Level B evidence (86, 87). However, recent cross-sectional surveys have not established a clear relationship between riboflavin intake and the prevalence of migraine in adults (69, 88).

Coenzyme Q10, also known as ubiquinone, is involved in the electron transport chain and aerobic respiration in the mitochondria of eukaryotic cells, playing an essential role in proton translocation and electron transport (89). Coenzyme Q10 acts as an activator of cellular respiratory metabolism, an antioxidant, and a non-specific immune enhancer. It is involved in mitochondrial oxidative phosphorylation, indirectly regulates extra-mitochondrial metabolic pathways, and protects cells against excessive ROS generation (90). Coenzyme Q10 also exhibits anti-inflammatory effects, reducing serum calcitonin gene-related peptide (CGRP) and tumor necrosis factor-*α* levels (91). Given its anti-inflammatory, antioxidant, and bioenergetic properties, coenzyme Q10 plays a key role in energy production pathways in the brain, and its deficiency can impair mitochondrial function (73). Coenzyme Q10 supplementation has been recommended as a safe and effective preventive therapy for migraine (74). Clinical trials have demonstrated that supplementation with coenzyme Q10 reduces the severity, frequency, and duration of migraine attacks, with effects typically observed within 4 weeks (92–94). It has shown favorable prophylactic effects in children aged 5–10 years with migraine and is well tolerated, with few side effects and no serious adverse events reported during long-term use (95, 96). However, while systematic reviews and meta-analyses affirm the role of coenzyme Q10 in reducing the frequency of migraine attacks, some controversy remains regarding its effects on headache severity and duration (97, 98). The combination of energy-modulating drugs is also of interest, with coenzyme Q10 combined with riboflavin or levocarnitine showing greater efficacy in alleviating migraine attacks (86, 99, 100). A prospective observational study suggested that the concomitant use of coenzyme Q10, magnesium, and feverfew may offer additional benefits for migraine management (101). These findings indicate that multi-targeted drug combinations focused on mitochondrial energy metabolism may be a promising direction for migraine prophylaxis.

ALA is an amphiphilic antioxidant that acts as a cofactor for pyruvate dehydrogenase and glycine decarboxylase. ALA directly or indirectly reduces oxidative stress in mitochondrial oxidative metabolism through its interaction with coenzymes such as triphenyl nitrate and nicotinamide adenine dinucleotide (102). Abnormally low levels of lactate have been observed in the serum of patients with migraine, suggesting that lactate could serve as a potential migraine biomarker (40, 103). ALA supplementation significantly reduces serum lactate levels and vascular cell adhesion molecule in female patients with migraine, suggesting that prophylactic ALA treatment may be beneficial for managing migraine (103). Additionally, ALA possesses anti-inflammatory and antioxidant properties. A clinical trial demonstrated that 3 months of ALA supplementation effectively improved oxidative, inflammatory, and emotional status in patients with migraine (104). A 6-month exploratory study also showed that ALA significantly reduced migraine days in patients with insulin resistance (99, 105). Beyond its effects in adult migraine, ALA was found to significantly reduce the frequency and severity of acute attacks in adolescent patients, along with lowering their serum CGRP levels (106). In conclusion, ALA supplementation may be considered a potential adjunctive treatment for patients with migraine (103, 107). Future clinical trials should further investigate its optimal dosage and administration schedule for migraine prophylaxis.

The ketogenic diet (KD) is a regimen that severely restricts carbohydrates and induces lipid metabolism and ketone body (KB) synthesis by mimicking a fasting state (108). KD has shown therapeutic potential in a variety of diseases, including obesity, epilepsy, metabolic syndrome, and Alzheimer’s disease (109). KBs serve as an alternative fuel source for the brain and may influence migraine pathophysiological mechanisms, such as mitochondrial function, oxidative stress, brain energy metabolism, and glutamate homeostasis (110–113). The effects of KBs on the central nervous system correlate with clinical improvements in migraine. KD has been found to improve migraine symptoms while significantly affecting cortical function-related potentials (114). KD plays an important role in reducing the frequency and severity of migraine attacks in both adolescents and adults (115), and it has also been shown to improve sleep disorders (116). As a result, KB supplementation may represent a potential prophylactic treatment strategy for migraine (117). A randomized controlled trial is currently investigating the safety and efficacy of exogenous KB salts, specifically 3-hydroxybutyric acid, for migraine prophylaxis (118). Additionally, recent studies suggest that both a 2:1 KD and a low-glycemic index diet may offer benefits for migraine treatment (119). However, future clinical studies are needed to monitor KB levels during KD and to explore the relationship between KB concentration and therapeutic efficacy.

## Conclusion

**Figure 2:**
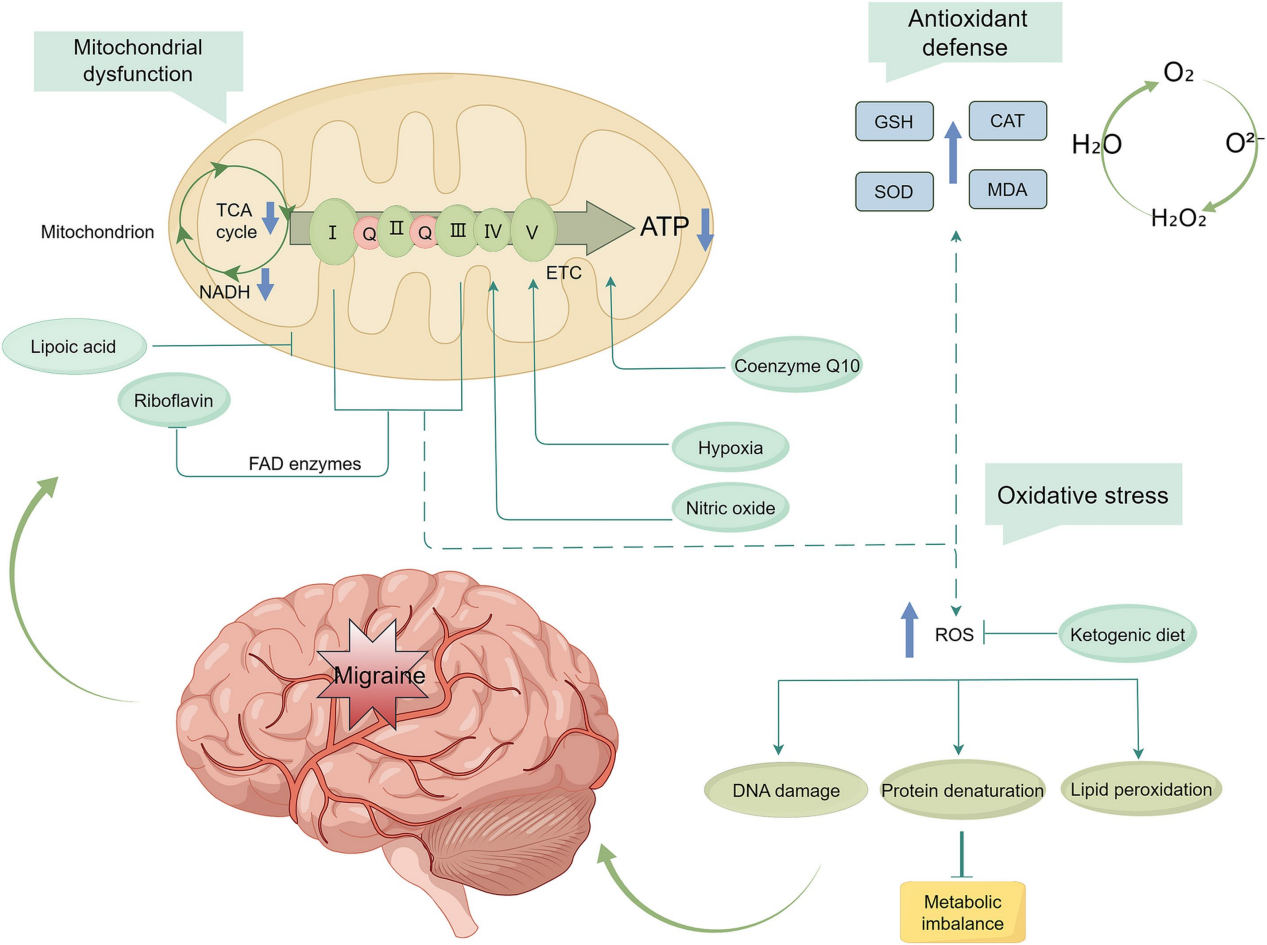
Migraine prophylactic targets for mitochondrial dysfunction. Nutrients play a protective role against migraine by modulating the mitochondria-energy production-oxidative stress pathway. These interventions help prevent excessive ROS production, thereby reducing oxidative stress and ameliorating energy deficits in the brain. TCA, tricarboxylic acid; ATP, adenosine triphosphate; NADH, nicotinamide adenine dinucleotide; ETC, electron transfer chain; GSH, glutathione; SOD, superoxide dismutase; CAT, catalase; MDA, malondialdehyde; ROS, reactive oxygen species.

Many migraine triggers are related to brain energy metabolism disorders. Inadequate energy supply increases susceptibility to CSD, and the brain energy deficit-mitochondrial-oxidative stress axis may represent a key pathway in migraine pathogenesis (Figure 2). Conditions such as diabetes mellitus, insulin resistance, high-density lipoprotein isoform alterations, fatty acid elongation enzyme activity, and metabolic syndrome have been associated with migraine. Additionally, metabolic changes in NAA/tCr, Cho/tCr, lactate, taurine, glutamate, GABA, and glycine levels have been observed in individuals with migraine. Patients with migraine also present with elevated oxidative stress and weakened antioxidant defenses. Nutrients may help improve mitochondrial function, thereby alleviating brain energy metabolism deficits and oxidative stress in migraine. Current prophylactic treatments targeting mitochondrial function and energy metabolism include riboflavin, coenzyme Q10, alpha-lipoic acid, and ketogenic ketones. However, the level of evidence supporting these treatments remains limited.
